# Detection and Genotyping of Human Adenoviruses in Wastewater from Bulgaria by Targeted Surveillance, In Vitro Cultivation, and Whole-Genome Sequencing

**DOI:** 10.3390/biology15151285

**Published:** 2026-08-04

**Authors:** Sergei Ivanov, Zoltan Urshev, Anton Hinkov, Petya Angelova, Kalina Shishkova, Stoyan Shishkov

**Affiliations:** 1 Group: “Microbiological Risks in the Environment”, Project SUMMIT 3.2.4., Faculty of Biology, University of Sofia “St. Kl. Ohridski”, 1164 Sofia, Bulgaria; sivanov714@abv.bg (S.I.); sshishkov@biofac.uni-sofia.bg (S.S.); 2 RnD Department, LB Bulgaricum EAD, 1407 Sofia, Bulgaria; zoltan_urshev@yahoo.com; 3 Laboratory of Virology, Faculty of Biology, University of Sofia “St. Kl. Ohridski”, 1164 Sofia, Bulgaria; pjangelova@uni-sofia.bg (P.A.); k_shishkova@biofac.uni-sofia.bg (K.S.); 4 Centre of Competence “Sustainable Utilization of Bio-Resources and Waste of Medicinal and Aromatic Plants for Innovative Bioactive Products” (BIORESOURCES BG), 1000 Sofia, Bulgaria

**Keywords:** wastewater-based epidemiology, human adenovirus, cell culture isolation, TaqMan qPCR, genome library preparation, shotgun sequencing, Bulgaria

## Abstract

Adenoviruses are detected in wastewater at significantly higher frequencies than most other enteric viruses. The aim of this study was to determine the abundance of human adenoviruses in samples collected from wastewater treatment plants in Sofia, Plovdiv, Burgas, and Varna using quantitative PCR (qPCR). During the study period in 2024, one or two peaks in adenovirus levels were detected in each city, with viral loads ranging from 10 to 30,000 genome copies/mL. Selected wastewater samples from Sofia were used to inoculate three different cell lines. Productive adenovirus replication was observed only in the Vero E6 cell line. Shotgun sequencing of DNA extracted from infected cells enabled the assembly of six complete adenovirus genomes from three qPCR-positive samples. Taxonomic analysis identified viruses belonging to the *Mastadenovirus adami*, *Mastadenovirus blackbeardi*, *Mastadenovirus caesari*, *and Mastadenovirus dominans* species, with the predominant genotype (three of six genomes) showing the highest similarity to HAdV-A31. Comparative genomic analysis provided evidence of molecular evolution driven by multiple recombination events. The recovered genomes contained hypervariable regions composed of genetic elements closely related to genotypes previously reported from different geographical regions. Overall, the integrated approach employed in this study demonstrates the value of combining wastewater surveillance, virus isolation, and whole-genome sequencing to monitor adenovirus circulation, characterize dominant variants, and assess their potential clinical significance.

## Simple Summary

Wastewater-based epidemiology (WBE) is an effective tool for monitoring infectious diseases because it enables the detection and characterization of viruses circulating within a community through wastewater analysis. It has been estimated that more than 90% of the global population is infected with at least one type of human adenovirus (HAdV) during their lifetime. Human adenoviruses can cause gastroenteritis, respiratory infections, and infections of the eyes, ears, and throat, and are detected in wastewater more frequently than many other human viruses. In this study, we monitored HAdV levels over a seven-month period in 2024 in the four largest cities in Bulgaria (Sofia, Plovdiv, Burgas and Varna). Quantitative PCR (qPCR) identified one or two peaks in adenovirus concentrations in each city, consistent with localized outbreaks. Virus isolation in cell culture enabled the recovery of infectious adenoviruses from wastewater samples. Whole-genome sequencing showed that the predominant isolates from Sofia belonged to *Mastadenovirus adami*, genotype A31. Surprisingly, the new adenoviruses from Sofia resemble the literary character of the novel Frankenstein, and are assembled from genetic elements of similar viruses found in different parts of the world. In conclusion, this study provides clear scientific evidence for the presence of novel adenovirus variants during periodic outbreaks of adenoviruses in urban populations, a fact that must be addressed by the Bulgarian public health system.

## 1. Introduction

Viruses are ubiquitous pathogens that are typically present at high concentrations in wastewater. Their taxonomic diversity generally reflects the epidemiological status of the human population contributing to the wastewater [1]. The isolation and identification of viral pathogens from wastewater provide valuable insights into the distribution and genetic diversity of viruses circulating within human populations. Most of the viruses detected are associated with human or animal diseases. During outbreaks, the predominant viral strains circulating in the affected population can also be identified in wastewater. Their detection and quantification enable the assessment of seasonal patterns of virus circulation [2] and support the evaluation of potential public health risks [3,4]. The presence of potentially pathogenic viruses in wastewater remains a public health concern because contaminated water may serve as a source of transmission for waterborne viral pathogens [5]. For example, numerous outbreaks of viral diseases have been linked to exposure to contaminated recreational waters [6,7], highlighting the need for routine and effective water quality monitoring. However, not all viral particles detected and quantified in wastewater are viable or infectious. Molecular methods such as qPCR may also detect non-infectious viral nucleic acids (“molecular corpses”), potentially overestimating the abundance of infectious viruses and, consequently, the associated public health risk. Cell culture remains the gold standard for determining viral infectivity, as it demonstrates the presence of viable viruses capable of replication and, therefore, of causing infection. In addition, virus isolation in cell culture often provides sufficient viral material for whole-genome sequencing and other downstream analyses of viral biology. Finally, the efficiency of wastewater treatment processes in reducing viral loads can be evaluated by assessing the infectivity of viruses recovered from treated effluent.

Viruses of the *Adenoviridae* family relevant to human pathology belong to the genus *Mastadenovirus*. The genus includes seven species (*adami*, *blackbeardi*, *caesari*, *dominans*, *exoticum*, *faecale*, *russelli*), with several serotypes assigned to each species. The family representatives are nonenveloped (without an outer lipid bilayer) with an icosahedral nucleocapsid containing a double-stranded, linear and non-segmented DNA genome (around 33–34 kb in length) [8]. Human adenoviruses are spread via respiratory droplets and by fecal–oral routes. They can cause respiratory diseases, gastroenteritis, conjunctivitis, tonsillitis, ear infections, or croup [9,10]. Due to the extremely broad spectrum of adenoviral pathologies, they are often confused with other pathogens, often bacterial, for example in upper respiratory tract infections [11]. Several studies have estimated that more than 90% of the human population worldwide is seropositive for one or more of the human adenovirus genotypes [12]. Mass infection, often in childhood, has long-term chronic consequences for human health. Adenoviruses can remain latent in the lymphoid tissue and reactivate when the immunological status of patients changes, for example during stress, severe illness, or advanced age. This leads to sudden and “causeless” chronic inflammations often confused with pathological manifestations with similar symptoms [13].

There are assumptions, but also evidence, that adenoviruses are associated with a number of socially significant diseases such as chronic obstructive pulmonary disease, obesity, in organ transplantation, or in patients on chemotherapy [14].

Adenoviruses are excreted for prolonged periods by infected persons at high concentrations of up to 10^11^ particles/g stool [2]. They occur at significantly higher frequencies in wastewaters than other enteric viruses [15]. In some studies, adenoviruses have been found in over 90% of wastewater samples [16]. They are the only enteric viruses with a double-stranded DNA genome, and thus they are relatively more stable and persistent in aquatic environments compared to most enteric viruses possessing an RNA genome [7]. Adenoviruses are unusually stable to chemical or physical agents and adverse pH conditions, allowing for prolonged survival outside of the body [17]. Because of this, they have been recommended as index organisms for viral pathogens in wastewater since they fit most of the requirements of an ideal indicator for anthropogenic contamination.

Wastewater adenovirus surveillance relies on contemporary molecular methods. One example is conventional PCR of adenovirus-specific gene targets, followed by sequencing to identify the particular viral species [16]. Universal quantitative PCR (qPCR) with TaqMan chemistry has been suggested to detect and quantify all adenoviruses in wastewater with a sensitivity of 15 copies/run [18]. Furthermore, methodology has been upgraded, as in the case of integrated cell culture-PCR (ICC-PCR), by combining qPCR with limited cultivation on cell lines to assess the type and viability of the detected adenoviruses in wastewater [17].

Human adenoviruses represent a significant public health concern, which prompted their inclusion in the national wastewater surveillance program. Therefore, the first objective of the study was to monitor temporal changes in adenovirus levels throughout 2024 in the four largest cities in Bulgaria. However, the study goes far beyond simple qPCR quantification of adenoviruses in wastewater, as it explores the necessary methodology and the depth that can be acquired in the identification of the viable adenovirus species in the samples and the assembly and analysis of complete adenoviral genomes.

## 2. Materials and Methods

### 2.1. Sampling and Processing of Wastewater

The sampling program includes the wastewater treatment plants of the capital Sofia, as well as the next most populous cities–Plovdiv, Varna and Burgas. Together, they represent almost 35–40% of the population of Bulgaria. Over a period of about 7 months (June–December 2024), 42 samples were collected, including 18 from Sofia, 10 from Plovdiv, and 7 each from Varna and Burgas [19]. One liter of wastewater was collected from each location, transported immediately to the laboratory in a refrigerator, and processed for up to a maximum of 24 h. The wastewater samples were first placed on a shaker at room temperature for 45 min. Then they were filtered through 4 layers of gauze, and 400 mL of the filtrate was centrifuged at 7000× *g*/45 min to remove bacteria. The supernatant was split into two halves of 200 mL, and each half was saturated to 10% PEG 8000 and 0.5 M NaCl, incubated for 2 h at 6 °C, and centrifuged at 13,500× *g*/60 min.

The precipitate from the first portion containing the concentrated viral fraction was resuspended in Viral Transport/Storage Medium (HiMedia, Maharashtra, India) and stored long-term in liquid nitrogen. After further processing, it was used for inoculation of cell lines. The precipitate from the second portion of 200 mL wastewater was resuspended in DNA/RNA Shield™ (Zymo, Research, CA, USA), and DNA was isolated with Quick DNA/RNA^TM^ viral kit (Zymo, Research, CA, USA). The purified DNA was stored at −20 °C and used for qPCR. With this protocol, the viruses in the wastewater samples were concentrated 1600 times.

### 2.2. Cell Lines

We used the Vero (Verda reno) E6 (C1008, ATCC CRL-1586, Manassas, VA, USA) cell line purchased from the American Type Culture Collection (ATCC)–USA, the BGM (Buffalo Green Monkey) cell line purchased from Cytion CLS Cell Lines Service GmbH–Heidelberg, Germany, and the MDBK (Madin-Darby Bovine Kidney) (ATCC, No. CCL-22) cell line purchased from the ATCC–USA. All cell lines were cultured at a temperature of 37 °C and 5% CO_2_ (CO_2_ incubator, BINDER Inc., Bohemia, NY 11716, USA) in Dulbecco’s Modified Eagle Medium (DMEM) (Sigma-Aldrich, Merck-Germany, 64293 Darmstadt, Germany) (enriched with 10% fetal calf serum (FCS) (Sigma-Aldrich, Merck-Germany, 64293 Darmstadt, Germany), 3 g/L glucose, 1.5 g/L NaHCO_3_, 20 mM HEPES buffer (Sigma-Aldrich, Merck-Germany, 64293 Darmstadt, Germany), 2 mM L-Glutamine and 8 g/L Gentamicin (Sigma-Aldrich, Merck-Germany, 64293 Darmstadt, Germany)).

### 2.3. Isolation of Viruses of the Adenoviridae Family

The samples containing the PEG-concentrated viral fraction were pre-diluted fivefold with DMEM maintenance medium supplemented with 4% FBS and filtered through a Millipore filter (0.22 µm) (Sigma-Aldrich, Merck-Germany, 64293 Darmstadt, Germany). The cell lines were cultured in a 24-well plate (Corning, New York, NY 14831, USA) and inoculated with the resulting filtrate (0.3 mL per well). After 2 h at a temperature of 37 °C and 5% CO_2_ (CO_2_ incubator, BINDER Inc., NY 11716, USA), maintenance medium was added (0.5 mL per well). The appearance of cytopathic effect (CPE) was monitored each day for up to 5 days, after which the plates were frozen at −80 °C (Ultra-low Temperature Freezer, BINDER Inc., NY 11716, USA). In the absence of CPE, up to 3 blind passages were performed, and the cell lysate in the maintenance medium was tested by TaqMan qPCR for the presence of the isolated virus. When CPE occurred and viral presence was proved by TaqMan qPCR, the culture medium from the cell lysate was used to inoculate a cell culture flask (25 cm^2^) (Corning, New York 14831, USA) to increase viral yield. It was stored at −80° C (Ultra-low Temperature Freezer, BINDER Inc., NY 11716, USA) for subsequent use.

### 2.4. Pan-Adenovirus qPCR

Virus detection was performed with TaqMan qPCR using a laboratory-developed protocol on a CFX Opus 96 instrument and a SsoAdvanced probes™ qPCR mix (BioRad, Hercules, CA 94547, USA). Typically, the reactions were set up as multiplex, combining the adenovirus test with quantification of other groups of viruses, including an internal control to detect potential inhibition. The pan-adenovirus primers and probe used in the study were as designed by [18]. An artificially synthesized oligonucleotide was used as a positive control, comprising a sequence within the hexon gene of the reference genome for *Human mastadenovirus blackbeardi* type 1 (HAdV B1) (GenBank Acc. No. NC_011203:18428-18579). Serial dilutions of the same oligonucleotide with known concentration were used to generate a standard curve for calculating the copy number of adenoviral DNA targets in the samples. A 109 bp oligonucleotide with a randomly generated sequence that showed no match in the GenBank nucleotide database was used as an internal control to demonstrate the absence of inhibition of the qPCR reaction. The reactions were performed in a total volume of 20 μL with initial denaturation at 95 °C for 2 min, followed by 40 cycles of 95 °C for 10 s and 60 °C for 20 s. The results were processed with BioRad CFX Maestro software, v.2.3. Each value in the figures is the average of at least three measurements ± standard error. The sequence of all oligonucleotides used in the study is presented in Table S1 (Supplementary Materials).

### 2.5. Library Preparation and Shotgun Sequencing

Viral DNA was isolated from Pan-adenovirus qPCR-positive cell cultures. A freeze-thaw cycle for cell destruction was applied. About 0.3 mL of the cell lysates was centrifuged at 5000× *g*/10 min to pellet cell debris. The supernatant was processed according to the protocol of the MagListi 5 M Viral DNA/RNA kit (Bioneer, Republic of Korea), and finally the purified DNA was eluted from the magnetic beads with 60 µL of 10 mM Tris-HCl buffer, pH 8.0. The DNA content (Qubit dsDNA HS) and the ratios 260/280 nm and 260/230 nm were measured (DS-11FX+ DeNovix Inc., Wilmington, DE, USA). If the sample passed QC, the library preparation for shotgun sequencing was continued. In the intermediate stages, the DNA was stored at −20 °C in Eppendorf^®^ DNA LoBind^®^ Microcentrifuge Tubes.

The QIAseq FX DNA Library UDI-A Kit HT (Qiagen, Hilden, Germany) and the corresponding procedure were used to prepare the library. The input samples contained between 10 and 100 ng of DNA. The enzymatic fragmentation was adjusted to obtain oligonucleotides with a length of 300 bp. Finally, the indexed library was amplified by PCR and purified and size-selected twice with QIAseq beads. The concentration of the library was measured with the QIAseq Library Quant Assay Kit (Qiagen). Premade libraries of individual samples were pooled and sent to the Novogene (Cambridge, UK) company. Novogene’s sample requirements were met, confirmed by preliminary QC. Sequencing was performed on the NovaSeq X Plus Series platform with PE150. Each cell culture sample was set for 5 GB sequencing depth. After analysis, the raw data was initially assessed by Novogene data quality control.

### 2.6. Bioinformatics

All bioinformatic analyses were performed using the online Galaxy platform [20]. Raw pair-end reads were preprocessed with the FastP software, v.1.0.1 [21] and assembled with Megahit v.1.2.9 [22]. The assembled sequences that showed >70% similarity with known adenoviral sequences were extracted with the BLAST software v.2.16.0 search program [23]. The terminal sequences and the overall size of the assembled adenoviral sequences were manually inspected to verify the completeness of the genome. The genomes were annotated using Prokka v.1.14.6. [24] and manually curated against reference genomes. The obtained adenoviral sequences were deposited in GenBank under the accession numbers PX951442, PX951443, and PZ023989-023992.

## 3. Results

### 3.1. Screening of Wastewater Samples for Adenoviruses

**Figure 1:**
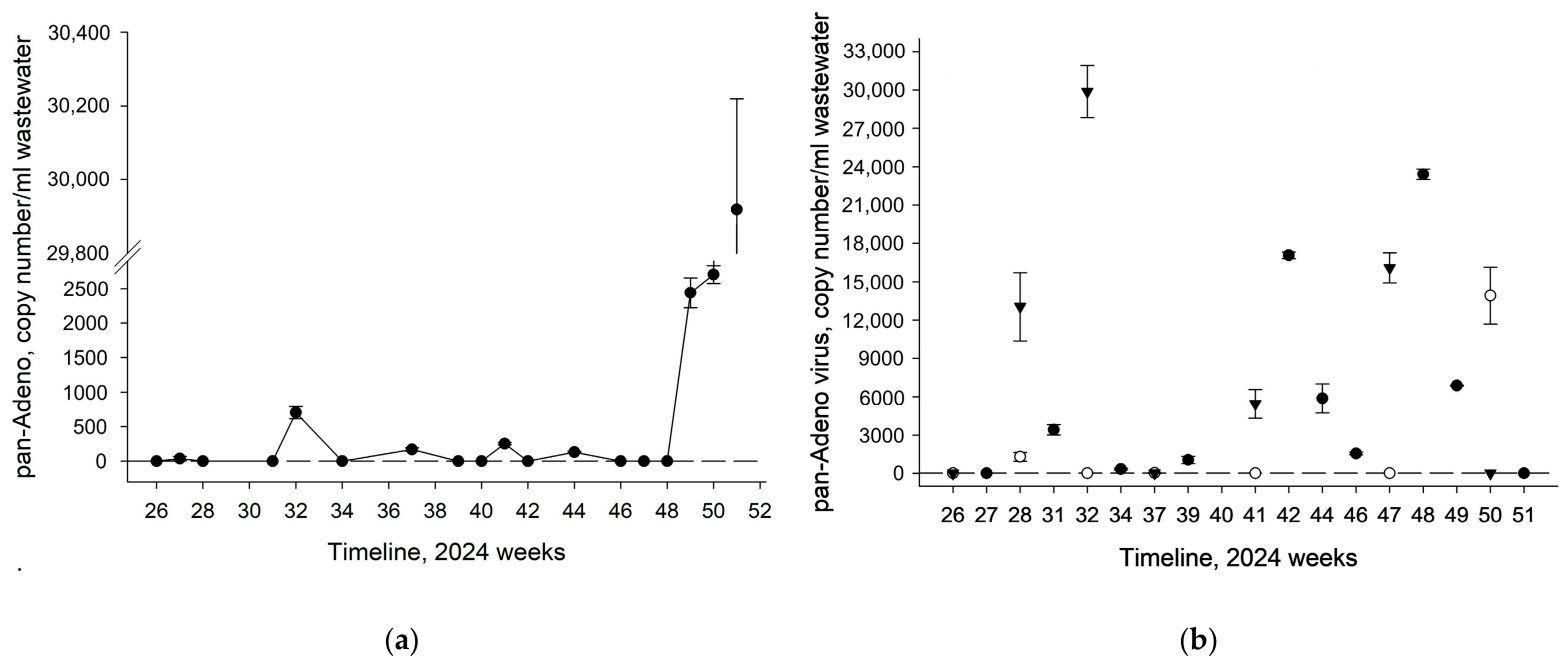
Monitoring of adenovirus levels in wastewater of Sofia (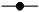) (**a**), and Plovdiv (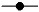), Varna (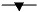) and Burgas (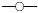) (**b**), from June to December 2024. Sampling was carried out from the inlet of wastewater treatment plants in the respective cities. Data are presented as number of viral target copies/mL raw wastewater ± standard error. LOD—limit of detection (-----).

Adenovirus surveillance is part of the national wastewater-based epidemiology (WBE) program, which also includes respiratory viruses such as SARS-CoV-2 and influenza viruses, as well as enteric viruses, including enteroviruses and hepatitis viruses, etc. [19]. Routine qPCR monitoring of wastewater began in June 2024; however, adenovirus had already been detected in the first pilot sample collected in February of the same year. A processed and purified viral concentrate from this initial positive sample collected in Sofia was cryopreserved in liquid nitrogen and subsequently used for the first successful isolation of adenovirus from wastewater within our project. By the end of 2024, a total of 18 wastewater samples from Sofia had been analyzed using a TaqMan qPCR assay, of which eight tested positive for adenovirus (Figure 1a). Two distinct peaks in viral concentration were observed: a smaller peak in August, reaching approximately 1000 genome copies/mL of wastewater, and a substantially larger peak in December, reaching approximately 29,000 genome copies/mL.

In Plovdiv, 8 of the 10 wastewater samples tested positive for adenovirus (Figure 1b). Similar to Sofia, a modest peak in adenovirus concentration was observed in August, followed by a more pronounced peak in October. In Varna and Burgas, seven wastewater samples were analyzed from each city during 2024 (Figure 1b). In Varna, adenovirus concentrations remained consistently high throughout the study period, reaching a maximum of approximately 30,000 genome copies/mL in August before declining sharply in the final sampling event in December. In contrast, the temporal pattern in Burgas resembled that observed in Sofia, with a marked increase in adenovirus concentration occurring only in December, which is the opposite of Varna and Plovdiv.

### 3.2. Cultivation of Adenoviruses on Different Cell Lines

Four samples from Sofia wastewater treatment plant with high copy number of measured *Adenoviridae* were selected for the cultivation trials. The samples were collected in February (late winter), July (two samples, collected at the beginning and end of the month), and December (early winter). Each sample was inoculated onto three cell lines: Vero E6, MDBK, and BGM. Infectious adenoviruses were successfully isolated from three of the four samples, but only in the Vero E6 cell line. Successful virus isolation and subsequent passages were confirmed by qPCR.

**Figure 2:**
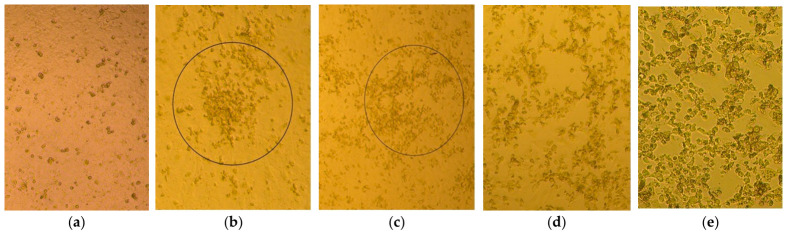
HAdV induced CPE in the Vero E6 cell line. (**a**) represents the cell control (mock-infected cells). Inoculation of the Vero E6 cell line with: (**b**,**c**) the third viral passage results in the appearance of small focal areas in the center of which there was a lytic field surrounded by morphologically altered cells (indicated by circles in the image); (**d**) the fourth viral passage increased the number and size of the affected areas of the cell monolayer; (**e**) the sixth viral passage results in the round-cell degeneration throughout a large portion of the monolayer while the remaining cells were rounded and reduced in size.

During the isolation of human adenoviruses from wastewater, all inoculated cell lines were monitored daily for up to five days for the development of cytopathic effects (CPE) before the 24-well plates were frozen. Cytopathic effects were observed exclusively in the Vero E6 cell line during the third viral passage (Figure 2).

Two blind passages, during which no visible cytopathic effects (CPE) were observed, were required for the isolated viruses to propagate to a higher titer into the Vero E6 cell line. This resulted in the development of characteristic morphological changes in the cell monolayer. Light microscopy of Vero E6 cells infected with the third viral passage revealed small focal lesions with a central lytic area surrounded by morphologically altered cells (Figure 2b,c). These changes were characterized by rounded, shrunken cells that had lost contact with neighboring cells of normal morphology, consistent with round-cell degeneration. In subsequent passages, both the number and the size of the affected areas increased, as evidenced by the expansion of the lytic foci and the increasing number of cells exhibiting round-cell degeneration (Figure 2d). By the sixth viral passage, the entire cell monolayer was affected, with no cells of normal morphology remaining (Figure 2e). Most infected cells had undergone lysis, whereas the remaining cells were rounded and markedly reduced in size. No cytopathic effects were observed in the MDBK or BGM cell lines throughout the three passages performed.

Wastewater typically contains very few infectious adenovirus particles. The first inoculation may therefore infect only a few cells without producing visible CPE. Blind passage further amplifies the virus that is still at levels below the threshold for microscopic detection. The use of up to three blind passages in permissive cell cultures is standard practice for recovering low-titer infectious adenoviruses from environmental samples [7,25,26]. This is consistent with ISO 15216-based (ISO 15216-1:2017; Microbiology of the food chain. Horizontal method for determination of hepatitis A virus and norovirus using real-time RT-PCR. Part 1: Method for quantification. International Organization for Standardization (ISO): Geneva, Switzerland, 2017) adaptations and World Health Organization environmental surveillance protocols, which typically limit culture amplification to three blind passages before concluding that infectious virus is not recoverable.

The freeze-thawed cells from the third viral passage of each inoculated cell line, together with the culture medium, were used for qPCR identification. Only the samples from the third viral passage of the Vero E6 cell line were CPE positive for human adenoviruses (Table 1).

### 3.3. Genotyping and Whole-Genome Sequencing of Cultured Adenoviruses

Lysates from infected cell cultures were processed and sent for shotgun sequencing-see “Library preparation and shotgun sequencing”. The resulting sequences were processed as described in the “Bioinformatics” section. As a result of the assembly process, a total of 7 large contigs were obtained from three separate cultured virus samples, qPCR positive with pan-adenovirus primers. Five of these contigs were identified as 100% complete genomes of novel adenovirus isolates. The remaining two represented two halves of a single viral genome. Additionally, taxonomic identification was performed with the software Kraken, v 1.3.1. Short fragments of adenoviruses, different from the obtained whole genomes, were discovered, but the quantity was insufficient to complete the assembly process.

From each of the samples with cultured viral concentrate from wastewater, between 1 and 4 genomes were assembled (Table 2). In the sample from February 2024, a single complete HAdV A (AdV-AS51) genome was found. The similarity to the reference genome (GenBank Acc. No. NC_001460) was weak, only 84.9%. AdV-AS51 was much closer (99.5%) to an adenovirus isolated 10 years earlier in the US (OQ518329.1).

From the wastewater sample from 09.07.2024, three adenoviral genomes were obtained: a complete HAdV B genome (AdV-BS31), a complete HAdV C genome (AdV-CS32), and a 98.6% complete HAdV A genome (AdV-AS33). The HAdV B and HAdV C isolates showed high identity with the respective reference genomes. The closest matches of AdV-BS31 and AdV-CS32, with about 99.9% identity, were adenoviruses from Korea and Russia, respectively (Table 2).

Two complete adenoviral genomes, one HAdV D (AdV-DS41) and one HAdV A (AdV-AS42), were assembled from data obtained for a sample from 30.07.2024. AdV-DS41 at 98% coverage was 96.8% identical with the respective reference genome (NC_010956). Evidence of recombination with other adenoviruses was detected in the AdV-DS41 genome. The annotated genomes of all new adenovirus isolates acquired under this project have been submitted to NCBI, with accession numbers shown in Table 2.

### 3.4. Evolution of HAdV A in Sofia-Evidence from Wastewater

**Figure 3:**
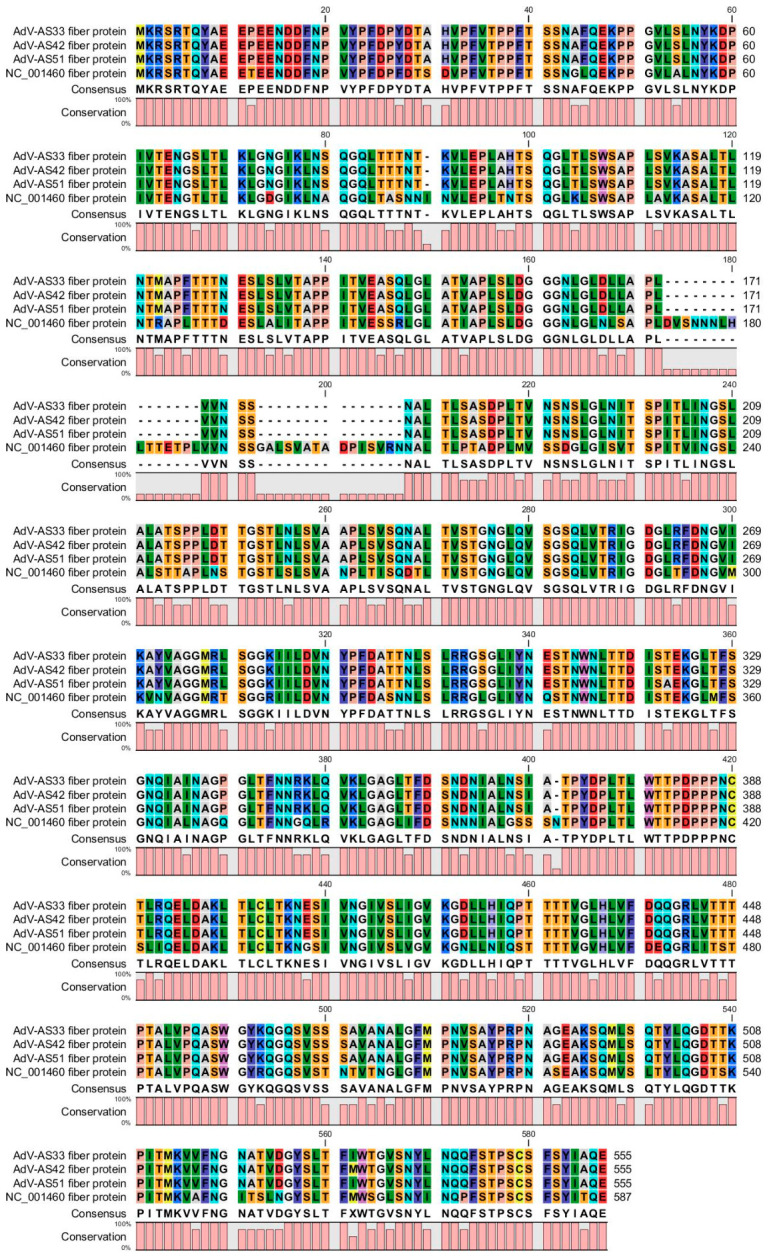
The amino acid sequence of the fiber protein of the three new isolates from Sofia compared to the HAdV A reference genome (NC_001460). All deviations of the amino acid sequence from the consensus are marked.

Viruses of the *Mastadenovirus adami* species, i.e., HAdV A, were successfully cultured and whole-genome sequenced from all of the three processed wastewater samples. A logical assumption is that this was the most widespread adenovirus in wastewater of Sofia during the monitored period. All identified HAdV A isolates belong to genotype A31, see Table 2. The three isolates, AdV-AS33, AdV-AS42 and AdV-AS51, showed very high identity to each other. Analysis of the amino acid sequences of their structural proteins revealed substantial differences in the fiber protein of the three isolates in comparison to the reference HAdV A genome (NC_001460) (Figure 3). With a total length of 587 amino acids, the fiber protein of AdV-AS33, AdV-AS42, and AdV-AS51 was shorter by 32 amino acids, including two regions with deletions of over 10 amino acids. In addition, 104 nonsynonymous substitutions were identified in comparison with the reference genome. A total of 136 amino acid substitutions were present in the fiber protein of the isolates from the wastewater of Sofia, which is 23.2% of its length. Of these 136 substitutions, only two were different between the three new isolates-positions 353 and 562. The mutation profile in the fiber protein of AdV-AS33, AdV-AS42, and AdV-AS51 is evidence that they probably belong to a new strain of *Mastadenovirus adami*, which persisted in the population of the capital Sofia for the duration of the monitored period.

## 4. Discussion

Wastewater-based epidemiology (WBE) is an approach that can provide comprehensive real-time health information for communities. WBE is an integrated technique related to the extraction, analysis, data processing, and interpretation of targets (so-called biomarkers) excreted with feces/urine in wastewater [27,28]. WBE serves as a practical and cost-effective surveillance method, offering real-time insights that can act as an early warning system for emerging outbreaks, help monitor trends in current epidemics, and support predictions about future disease spread [29,30]. This technique should be flexible, cost-effective, and scalable. As a public health tool, WBE has demonstrated a clear link between viruses detected in the environment and those identified in clinical cases within the same region and time period. Often, the most common virus type found in patients is also the dominant type detected in wastewater during that year. Compared with individual patient testing, WBE offers several advantages, including lower costs and the ability to gather large-scale population data while enabling earlier detection of infectious threats [31,32].

Human adenoviruses are commonly found in wastewater and are recognized for their strong resistance to inactivation. Unlike enveloped RNA viruses (such as SARS-CoV-2), adenoviruses lack a lipid envelope, making them extremely resistant to temperature fluctuations and chemical purification [33,34]. Due to this high environmental stability, HAdVs are regularly detected in both untreated and treated wastewater (effluent). Human adenoviruses are recognized as an ideal index organism for viral contamination of human origin [7,35]. Monitoring for adenoviruses in wastewater of large Bulgarian cities showed a profile similar to most analogous studies. Out of every 3 tested samples, 2 proved positive for the pathogens (see Section 3). In terms of detection frequency in Bulgarian wastewater, adenoviruses are only surpassed by noroviruses and some specific markers of fecal contamination such as CrAssphage (unpublished results). Surprisingly for us, each individual city has its own dynamics of adenovirus levels measured in wastewater, with quantities varying greatly, from 10 copies/mL to 30,000 copies/mL. In the summer, the largest peak was established in the resort city of Varna (Figure 1b), while in the capital Sofia, an explosive increase in the concentration of the virus was detected in December (Figure 1a). The trend is generally warm season/cold season extremum, with some specificity for each city. The sharp increases in the levels of human adenoviruses in wastewater directly reflect changes in the intensity of infections among the population and are characteristic of this pathogen. A typical example is the peak of adenoviruses detected in Milan’s wastewater in the winter of 2021/2022, in which the HadV-F41 strain was identified, responsible for the increased admissions to local hospitals of children with acute respiratory and gastrointestinal symptoms [36]. Another example of how wastewater-based epidemiology (WBE) can help estimate the true infection burden of a pathogen during an outbreak, particularly when clinical surveillance is limited, is a study in which wastewater monitoring was used to track *Human mastadenovirus faecale* types 40 and 41 (HAdV F40 and F41) in Sweden. During the spring of 2022, 12 cases of non-A-to-E hepatitis were reported in Sweden, two of which were severe enough to require liver transplantation. Clinical testing for adenoviruses in Sweden is primarily performed on hospitalized and critically ill patients; therefore, positive clinical samples do not accurately reflect the disease burden in the general population. Consequently, the true weekly prevalence of HAdV F infections in Stockholm remained unknown.

Analysis of samples collected from wastewater treatment plants enabled researchers to assess the overall prevalence of HAdV F in the Stockholm population, including undiagnosed and non-hospitalized cases. The HAdV F outbreak detected through wastewater surveillance suggested a substantially higher infection burden than that indicated by reported clinical cases. Although the limited availability of clinical data prevented detailed statistical analyses, wastewater monitoring provided an alternative approach for estimating the true infection burden of HAdV F. Given the restricted clinical surveillance of HAdV F40/41 in Sweden, this method proved particularly valuable for assessing the extent of virus circulation during the outbreak [37]. However, the epidemiological peaks identified in our study did not correlate with officially reported infectious disease statistics in Bulgaria. This discrepancy is likely attributable to the very limited routine testing of patients for adenovirus infections in the country. The summer peak of adenoviruses in the wastewater of a resort city like Varna coincides with a mass incidence of gastrointestinal and “summer flu”-like symptoms, and it is possible that this virus is the cause of the indication. In Sofia, the warm season extremum of HAdV was of much lower intensity than in Varna. Unlike Varna, in Sofia in the summer of 2024 a peak in the concentration in wastewater and the morbidity of patients with SARS-CoV-2 was noticed [19]. We hypothesize that in the summer of 2024 the two cities radically differed in the profile of the viruses prevailing in the population. The human adenovirus was predominant in the population of Varna, but in Sofia it was SARS-CoV-2. Both viruses cause “summer flu”-like symptoms during the summer months; however, adenovirus infections in Varna may have additionally contributed to gastrointestinal manifestations. Nevertheless, this hypothesis cannot be confirmed without systematic clinical testing and surveillance of patients.

It is reasonable to assume that the predominant adenoviruses in the populations may be specific to individual geographic locations, and may also exhibit seasonal variation. Molecular genetic testing using qPCR and pan-adenovirus primers quantifies the total adenoviral load in wastewater but does not differentiate between individual species, genotypes, or strains. In parallel with the other experimental approaches, we attempted direct metagenomic profiling of viruses present in wastewater (unpublished data). Although partial sequences from different adenoviruses were detected, the sequencing depth was insufficient for reliable genotyping and the assembly of complete genomes. Similar limitations have been reported by other researchers [38]. Viral genome reconstruction can be improved using Amplicon-based whole-genome sequencing or Hybrid-capture enrichment approaches [39]; however, these methods may still result in incomplete genome recovery and can be affected when substantial genetic divergence or mutations are present relative to the reference genome.

To characterize adenoviruses present in wastewater, we applied the gold-standard approach of virus cultivation in cell lines. Wastewater concentrates were inoculated onto three cell lines, each of which expresses the Coxsackievirus and adenovirus receptor (CAR) and therefore has the potential to support adenovirus infection [40,41]. However, cytopathic effects (CPE) were observed exclusively in the Vero E6 cell line after the third viral passage. This finding was confirmed by positive qPCR results using pan-adenovirus primers, whereas the MDBK and BGM cell lines showed neither CPE development nor qPCR evidence of adenovirus replication.

Our results indicate that Vero E6 cells, although not routinely used for adenovirus isolation, may represent a useful addition to the panel of cell lines employed for the isolation and propagation of human adenoviruses. The isolation of human adenoviruses in cell cultures could be considered an important step toward identifying the predominant genotypes present in environmental samples, although this is a slow and time-consuming process. Nevertheless, the results obtained contribute significantly to ongoing efforts to monitor viruses in wastewater. This is also a necessary step for whole-genome sequencing, which would enable us to monitor the emergence of mutations and viral evolution. Furthermore, characterizing isolates from wastewater can help inform public health professionals about the dominant and emerging genotypes circulating within the population (leading to hidden epidemics in the human population), providing new insights into vaccine development and other prevention strategies [42].

The successful whole-genome sequencing of adenoviruses achieved in this study, following their propagation in the Vero E6 cell line, enabled the detailed identification and characterization of adenovirus species simultaneously present in wastewater. Three successfully cultivated isolates were subjected to shotgun sequencing, resulting in the assembly of six complete adenovirus genomes (Table 2). The three predominant strains identified in Sofia wastewater showed the highest similarity to the A31 genotype. Whole-genome analysis revealed that these three novel A31-like strains (PX905700, PZ023991/2 and PX951442) differ substantially from the reference *M. adami* genome (NC_001460) but are closely related to other isolates identified in geographically diverse locations worldwide. Additional analysis of the amino acid sequence of the defining fiber protein of A31 (for all three A31-like strains recovered from Sofia wastewater) found a 23.2% difference in length compared to the reference genome. Only 2-point mutations distinguished the three newly identified strains isolated from Sofia wastewater from each other (Figure 3). The fiber protein plays a critical role in adenovirus biology, as it mediates viral attachment to host cell receptors [43]. The substantial alteration in fiber protein length (a change equal to 23%) shared by all three A31-like strains recovered from Sofia wastewater may suggest the acquisition of novel tissue tropisms.

Table S2 deepens the characterization of the new A31-like strain AdV-AS42 (PX951442). The complete genome shows 99.9% nucleotide identity to an A31 strain previously isolated in Canada (MN901813). However, comparative analysis of the amino acid sequences of AdV-AS42 core genotyping proteins (DNA_polymerase, fiber, penton, and hexon) reveals a real surprise. These core proteins were not closely related to those of the Canadian A31 isolate (MN901813), but rather to proteins encoded by distinct adenovirus genomes. For example, the fiber protein sequence is 100% identical to an A31 strain isolated in Hannover (MZ983553). On the other hand, compared with the entire genome of AdV-AS42, the Hanover isolate (MZ983553) has a total coverage of only 9% and only 75.8% identity, respectively. In summary, the new isolate from Sofia wastewater, AdV-AS42 „stole” from the Hanover isolate (MZ983553) (Figure S1) the complete fiber protein, and an additional 94% of its penton protein, both with 100% sequence identity. Additionally, AdV-AS42 recombined 100% with the DNA polymerase sequence from another A31 strain (MZ983562). Thus, the AdV-AS42 genome appears to have been shaped by the acquisition and exchange of key genetic regions from closely related adenovirus lineages. Recombination is considered a major driver of molecular evolution in adenoviruses [44], allowing these viruses to generate genetic diversity through the exchange of large genomic regions rather than relying solely on the accumulation of point mutations.

Recombination predominantly affects hypervariable regions, particularly those encoding the penton, hexon, and fiber proteins. These genetic exchanges can alter the antigenic properties of recombinant adenoviruses and influence their tropism, including their preferred host-cell receptor interactions [45,46]. In the human population of Sofia, adenoviruses may constitute a diverse pool of related genotypes generated through homologous recombination involving closely related isolates from different geographical regions worldwide. This dynamic viral population continues to evolve through adaptation to human hosts and the acquisition of novel genetic elements from circulating adenovirus lineages in an increasingly interconnected world. However, the clinical significance and potential pathogenic consequences of these newly identified adenovirus strains remain to be determined.

Despite the large amount of data generated, our study has several limitations:

1. The sensitivity of virus detection in wastewater is highly dependent on the methodology and analytical platform used. In this study, we employed qPCR; however, under otherwise comparable conditions, droplet digital PCR (ddPCR) generally provides higher sensitivity and greater quantitative accuracy.
2. The results presented cover only the first seven months of monitoring. This observation period is relatively short, and some of the observed findings may reflect random variation rather than consistent epidemiological trends. Longer-term surveillance is needed to confirm these observations.
3. Successful isolation and cultivation of adenoviruses from wastewater is a rare event so far and may selectively favor particular viral strains. Therefore, additional evidence is needed to ensure that the adenovirus strains from wastewater propagated in cell culture accurately represent the predominant strains circulating in the human population.

## 5. Conclusions

This study presents the results of human adenovirus surveillance in major Bulgarian cities during 2024. To the best of our knowledge, this is the first study in Bulgaria to investigate adenoviruses in wastewater. Although limited data are available from clinical patients and no genotyping has been performed. We applied an integrated approach combining targeted qPCR analysis with the cultivation of selected wastewater samples in cell lines, followed by whole-genome sequencing of the recovered isolates. Our findings demonstrate that monitoring adenovirus levels in wastewater is an effective approach for assessing the epidemiological status of specific geographic locations/cities. Genomic characterization of cultured adenoviruses revealed that a group of closely related strains associated with genotype A31 predominated in the capital city, Sofia. This is clear scientific evidence for the presence of novel adenovirus variants during periodic outbreaks of adenoviruses in urban populations, a fact that must be addressed by the Bulgarian public health system. Further analysis indicated that these strains have evolved through recombination events involving genetic elements derived from adenoviruses identified in different countries of the world. The combined approach applied in this study enabled detailed characterization of the dominant human adenovirus variants in the local population. It delivered significantly more information than the classical targeted analysis of wastewater alone. Future implementation of metagenomic approaches would further complement this methodology by enabling broader characterization of the viral diversity present in wastewater.

## Figures and Tables

**Table 1:** Cultivation of three types of cell cultures with viral concentrates isolated from wastewater of the capital Sofia. The table summarizes the results, appearance of morphologically detectable cytopathic effect (CPE), and pan-adenovirus qPCR identification, after the first three passages.

Date (Month/Year)	Cell Lines Used in the Isolation of Viruses from the Family *Adenoviridae*								
	Vero E6			MDBK			BGM		
	CPE ^#^	qPCR Identification (Passage on Which It Was Carried Out)	First Passage of CPE ^#^ Observation/Hours After Inoculation	CPE ^#^	qPCR Identification (Passage on Which It Was Carried Out)	First Passage of CPE ^#^ Observation/Hours After Inoculation	CPE ^#^	qPCR Identification (Passage on Which It Was Carried Out)	First Passage of CPE ^#^ Observation/Hours After Inoculation
02/2024	(+) ^1^	(+) ^2^ (3)	3/72	n.d. *	(—) ^3^ (3)	n.d. *	n.d. *	(—) ^3^ (3)	n.d. *
07/2024 (first week)	(+) ^1^	(+) ^2^ (3)	3/72	n.d. *	(—) ^3^ (3)	n.d. *	n.d. *	(—) ^3^ (3)	n.d. *
07/2024 (last week)	(+) ^1^	(+) ^2^ (3)	3/72	n.d. *	(—) ^3^ (3)	n.d. *	n.d. *	(—) ^3^ (3)	n.d. *
12/2024	(+) ^1^	(+) ^2^(3)	3/72	n.d. *	(—) ^3^ (3)	n.d. *	n.d. *	(—) ^3^ (3)	n.d. *

**^#^**^1^ presence of CPE, ^2^ (+) a positive result from qPCR, ^3^ (—) negative result from qPCR. * n.d.—not detected.

**Table 2:** Description of the adenoviruses isolated after cultivation on cell lines. Regarding the processing of the new genomes, see section “Bioinformatics”. For each new genome, the corresponding NCBI accession number is presented. Analyses, including “percent Identity”, “query cover”, as well as the identification of closest genomes, were calculated using NCBI blastn.

Identification	Sampling	HAdV Type	Completeness (%)	Size (bp/Number) of Identified ORFs	GenBank Acc. Num.	Identity with Reference Genome (Acc. Num.)/(%). 100% Coverage	Identity with Closest Genome (Acc. Num.)/(%). 100% Coverage
AdV-AS51	02.02.2024	HAdV A	100	33,780/33	PX905700	NC_001460/84.9	OQ518329.1/99.5 HAdV-A31/USA10K2/2013
AdV-AS33	09.07.2024	HAdV A	98.6	33,645/31	PZ023991/2	NC_001460/84.9	MN901807.1/99.86 HAdV A31ONP09CU1DEC2015
AdV-BS31	09.07.2024	HAdV B	100	35,213/37	PZ023989	NC_011203/98.4	PX461458.1/99.99 HAdV B, KOR/GJ-2024-0701
AdV-CS32	09.07.2024	HAdV C	100	35,862/39	PZ023990	NC_001405/99.7	MZ151863.1/99.89 HAdV C/Novosibirsk/8.202V/2020
AdV-AS42	30.07.2024	HAdV A	100	33,723/33	PX951442	NC_001460/84.9	MN901813.1/99.99 HAdV-A31ONP19CU1NOV2016
AdV-DS41	30.07.2024	HAdV D	100	35,094/33	PX951443	NC_010956/96.8 Request coverage: 98%	LC066535.1/95.05 HAdV D, JPN/OIPH23/2011/82[P56H15F37]

## Data Availability

The data presented in this study are available in the article. Further information is available upon request from the corresponding author.

## Supplementary Materials

The following supporting information can be downloaded at: https://www.mdpi.com/article/10.3390/biology15151285/s1, Table S1: List of oligonucleotides (primers, probes, positive controls) used in the qPCR reaction for detection of Pan-adenovirus targets, as well as for inhibition control-NS150 artificial oligo; Table S2: Analysis of possible recombinant events in the genome of the new AdV-AS42 (PX951442); Figure S1: Graphical representation of the nucleotide sequences from MZ983553.1 (marked in red) that have recombined into the genome of the AdV-AS42 (PX951442) strain isolated from Sofia wastewater–processed by NCBI blastp.

## Abbreviations

The following abbreviations are used in this manuscript:

AA	Amino acid
Acc. Num.	Accession Number
BGM	Buffalo Green Monkey
bp	Base pair
CAR	Coxsackievirus and adenovirus receptor
CPE	Cytopathic Effect
DMEM	Dulbecco’s Modified Eagle Medium
DNA	Deoxyribonucleic Acid
FCS	Fetal Calf Serum
HAdV	Human Adenovirus
Ident.	Identity
MDBK	Madin-Darby Bovine Kidney
NCBI	National Center for Biotechnology Information
Nucl	Nucleotide
ORF	Open Reading Frame
PEG	Polyethylene Glycol
PCR	Polymerase Chain Reaction
ddPCR	Droplet digital Polymerase Chain Reaction
qPCR	Quantitative Polymerase Chain Reaction
RNA	Ribonucleic Acid
SARS-CoV	Severe Acute Respiratory Syndrome Coronavirus
Vero	Verda reno
WBE	Wastewater-based epidemiology
