# Supplementary material for: Detection and Genotyping of Human Adenoviruses in Wastewater from Bulgaria by Targeted Surveillance, In Vitro Cultivation, and Whole-Genome Sequencing
Source: Biology (Basel). 2026 Aug 4;15(15):1285. doi: 10.3390/biology15151285 (PMC13464886; doi:10.3390/biology15151285)
Supplement: Supplementary file 1 [file biology-15-01285-s001.zip › Table S1.pdf]

Table S1. List of oligonucleotides (primers, probes, positive controls) used in the qPCR reaction for detection of Pan-adenovirus targets, as well as for inhibition control - NS150 artificial oligo.

Types of PCR reaction	Primer/probe/control sequence	Sequence 5′-3′	Reference
Pan-adenovirus qPCR	HadV-F	gccccagtggtcttacatgcacatc	[18]
	Hadv-R	gccacggtggggtttctaaactt	
	HadV-probe	FAM- tgcaccagacccgggctcaggtactccga-BHQ1	
	HadV-positive control	catcgatgatgccccaatgggcatacatgcacatcgccggacaggatg cttcggagtacctcagtccgggtctggtgcagttcgcccgtgcaacaga cacctacttcagtatggggaacaaatttagaaaccccacagtggcgccc acccac	this sudy
Inhibition control	NS150-F	tttcaagtcggaccctgctc	this sudy
	NS150-R	gcatcggcagataaattcctacg	
	NS150-probe	Cy5-ggtgacctgtcggggttggtcca-BHQ3	
	NS150-template	aatcctttcaagtcggaccctgctcgcgaggtagttttacccggtgacct gtcggggttggtccataactggctgttgatacgtaggaatttatctgccg atgcaaact	
