# Supplementary material for: Detection and Genotyping of Human Adenoviruses in Wastewater from Bulgaria by Targeted Surveillance, In Vitro Cultivation, and Whole-Genome Sequencing
Source: Biology (Basel). 2026 Aug 4;15(15):1285. doi: 10.3390/biology15151285 (PMC13464886; doi:10.3390/biology15151285)
Supplement: Supplementary file 1 [file biology-15-01285-s001.zip › Table S2.pdf]

Table S2. Analysis of possible recombinant events in the genome of the new AdV-AS42 (PX951442).

Analyzed genome/separate gene	Type of sequence	Closest GenBank Acc. Num.	Cover (%)	Ident. (%)	Locus	Notes
PX951442/AdV-AS42	Nucl	MN901813.1	100	99.99		complete genomes
DNA_polymerase[1]	AA	MZ983562.1	100	100	UNA48224.1	
		MZ983603.1	100	99.83	UNA49618	
Fiber protein[1]	AA	MZ983553.1	100	100	UNA47923.1	PX905700.1 with identity of 99.64%
		MZ983601.1	100	99.82	UNA49555.1	
Hexon protein[1]	AA	PX905700.1	97	99.89	YDV08524.1	
		KF360047.1	98	96.21	AIK20560.1	
Penton protein[1]	AA	MZ983553.1	94	100	UNA47925.1	
		KF268119.1	94	99.80	AGT75506.1	
MZ983562.1 Human adenovirus 31	Nucl		9	75.75		complete genome[2]
MZ983603.1 Human adenovirus 31	Nucl		9	75.75		complete genome[2]
MZ983553.1 Human adenovirus 31	Nucl		9	75.80		complete genome[2]
MZ983601.1 Human adenovirus 31	Nucl		9	75.80		complete genome[2]
KF360047.1 Simian adenovirus ch1	Nucl		8	75.80		complete genome[2]
KF268119.1 Human mastadenovirus A	Nucl		9	75.75		complete genome[2]

[1]Extracted from PX951442/AdV-AS42 amino acid sequences of the indicated proteins were blastp to identify the closest analogues in the NCBI library.

[2]Comparison of the whole nucleotide sequence of the corresponding strain from this row of the table with PX951442/AdV-AS42.
